# Ultra-early neurological deterioration following a brain arteriovenous malformation rupture

**DOI:** 10.3389/fneur.2024.1432687

**Published:** 2024-08-23

**Authors:** Eimad Shotar, Pierre-Marie Chiaroni, Idriss Haffaf, Jonathan Cortese, Alice Jacquens, Lorenzo Garzelli, Julien Allard, Mahmoud Elhorany, Caroline Amouyal, Bertrand Mathon, Aurélien Nouet, Kévin Premat, Stéphanie Lenck, Nader-Antoine Sourour, Vincent Degos, Frédéric Clarençon

**Affiliations:** ^1^Neuroradiology Department, Pitié-Salpêtrière Hospital, Paris, France; ^2^Sorbonne Université, INSERM, Institut de la Vision, Paris, France; ^3^Sorbonne Université, Paris, France; ^4^Neurosurgical Anesthesiology and Critical Care Department, Pitié-Salpêtrière Hospital, Paris, France; ^5^Department of Neurology, Faculty of Medicine, Tanta University, Tanta, Egypt; ^6^Neurosurgery Department, Pitié-Salpêtrière Hospital, Paris, France

**Keywords:** arteriovenous malformation, hemorrhage, deterioration, hydrocephalus, rupture

## Abstract

**Purpose:**

This study aims to explore the impact of ultra-early neurological deterioration (U-END) on the outcome (mortality and poor neurological status) following a brain arteriovenous malformation (BAVM) rupture and identify determinants of U-END.

**Methods:**

Patients with BAVM ruptures admitted to a single tertiary care center were retrospectively reviewed. U-END was defined as a worsening by two or more points on the Glasgow Coma Scale (GCS). U-END was tested as a potential predictor of in-hospital mortality and poor outcomes. Univariate and multivariate analyses were performed to identify determinants of U-END. Patients with U-END were also matched and compared with BAVM rupture controls presenting with a GCS close or equal to either their initial or their lowest GCS.

**Results:**

A total of 248 patients with BAVM ruptures met the inclusion criteria, with 39 (15.7%) patients presenting with U-END. U-END was not associated with and was not an independent predictor of in-hospital mortality (12.8 vs. 10.5% in the rest of the study population; *p* = 0.67) or poor outcomes (39.5 vs. 36.9%; *p* = 0.77). The only independent determinants of U-END were hydrocephalus (OR 2.6 [95%CI, 1.1–6.4]; *p* = 0.03) and intraventricular hemorrhage (IVH; OR 3.5 [95%CI, 1.1–11.7]; *p* = 0.04). When compared to the initial GCS control group, U-END patients more often presented with IVH (89.5 vs. 64.1%; *p* = 0.009) and hydrocephalus (73 vs. 38.5%; *p* = 0.003). When compared to the lowest GCS control group, U-END patients had lower early S100B serum levels (0.35 ± 0.37 vs. 0.83 ± 1; *p* = 0.009) and a lower rate of poor outcome (39.5 vs. 64.9%; *p* = 0.03).

**Conclusion:**

Ultra-early neurological deterioration in ruptured BAVMs did not result in increased mortality or poor outcomes and was most often related to IVH and hydrocephalus.

## 1 Introduction

Ultra-early neurological deterioration (U-END) has been associated with poor outcomes and increased mortality following intracerebral hemorrhage (1). Brain arteriovenous malformation (BAVM) rupture is the leading cause of morbidity, mortality, and social and healthcare burden in this disease (2–4). However, BAVM ruptures significantly differ from primary intracerebral hemorrhage (ICH) with respect to several clinical characteristics. Patients with BAVM ruptures tend to be younger, have lower pre-stroke and admission blood pressure, have a higher Glasgow Coma Scale (GCS) at admission, and are more likely to have an ICH in a lobar location (5). Moreover, ICH is absent in more than 20% of BAVM ruptures, and BAVM-related hemorrhage appears to have a better long-term outcome than spontaneous hemorrhage (5–7).

The primary objective of this study was to explore the impact of U-END on outcomes (mortality and poor neurological status) following BAVM rupture. The secondary objective was to identify determinants of U-END in this specific setting.

## 2 Materials and methods

### 2.1 Ethical statement

The institutional review board (IRB reference CRM-201-119) approved this study. The need for patients’ informed consent was waived. This study complies with the principles of the Declaration of Helsinki (1964).

### 2.2 Definitions

Ultra-early neurological deterioration was defined as a worsening by two or more points on the GCS in the prehospital setting or early post-arrival, with early post-arrival defined as within 48 h of admission as previously reported (1). In-hospital mortality was defined as death from any cause during the initial hospital stay. Poor outcome was defined as a modified Rankin scale score of ≥3 at 3 months or beyond following admission.

### 2.3 Patients

Records of patients with BAVM ruptures admitted to a tertiary care teaching hospital from 1 January 2005 to 31 January 2020 were retrospectively reviewed. Clinical, demographic, and imaging data were recorded. Post-embolization ruptures were excluded. Patients with an unknown initial GCS score or patients known to have deteriorated early before or upon admission but for which the extent of deterioration was not recorded were also excluded. Because BAVM patients can suffer multiple hemorrhages during their lifespan, patients could be admitted multiple times, having a different clinical severity profile for each rupture, with or without U-END. In-hospital mortality and neurological outcomes were recorded. ICH volume was measured using the ellipsoid volume approximation (ABC/2) method, in which A is the greatest diameter on the slice on which the hematoma appears the largest, B is the diameter perpendicular to A, and C is the approximate number of axial slices, with hemorrhage multiplied by the slice thickness, which has been validated in several large studies (8–10). Graeb score [an estimate of intraventricular hemorrhage (IVH) severity based on gross hemorrhage size] and Barrow Neurological Institute [BNI; an estimate of subarachnoid hemorrhage (SAH) severity] score were calculated as previously reported (11, 12). S100B protein is a biochemical marker of secondary neurological complications in neurocritical care patients (13). Early S100B protein serum elevation was defined as a maximal value within the first 48 h following the admission of >0.5 μg/L (S100B48max) (14). Assessment and correction methods of S100B data samplings were handled as previously described (14).

### 2.4 Statistical analysis

Patients with and without U-END were compared using univariate analysis and Kaplan–Meier survival analysis. Multivariate stepdown logistic regression was performed to determine whether U-END is an independent predictor of in-hospital mortality or poor outcome. Because this is not a broad exploratory analysis, only previously known predictors of in-hospital mortality and poor outcome in patients with BAVM rupture were included in the model (initial GCS ≤ 8, ICH volume, IVH, and early elevated S100B serum protein) (4, 14), in addition to U-END. Receiver operating characteristic (ROC) curves for in-hospital mortality and poor neurological outcome were drawn to compare initial GCS and the lowest early GCS as predictors of outcome. The area under the ROC curves (AUC) was calculated for the different endpoints and compared between the two GCS measures (initial and lowest). The lowest GCS was defined as the lowest grade reached by the patient before stabilizing or being sedated.

A multivariate stepwise logistic regression analysis was performed to identify determinants of U-END. Variables were included in the model if they were associated with U-END in univariate analysis with a *p* value of <0.1.

To refine the clinical description of U-END patients, two control groups were constituted and compared to the U-END subgroup: each U-END patient was randomly matched with a control patient having a GCS equal or nearly equal (±1 GCS point) to either his initial or his lowest GCS (the lowest GCS comparison group). Binary variables were compared using a Chi-square test, while means and medians were compared using a Student’s *t*-test and a Mann–Whitney test, respectively. The *p* values of <0.05 were considered significant. Statistical analyses were performed using MedCalc version 19.2.6 (Ostend, Belgium).

This study adheres to the STROBE guidelines for observational studies.

## 3 Results

**Figure 1:**
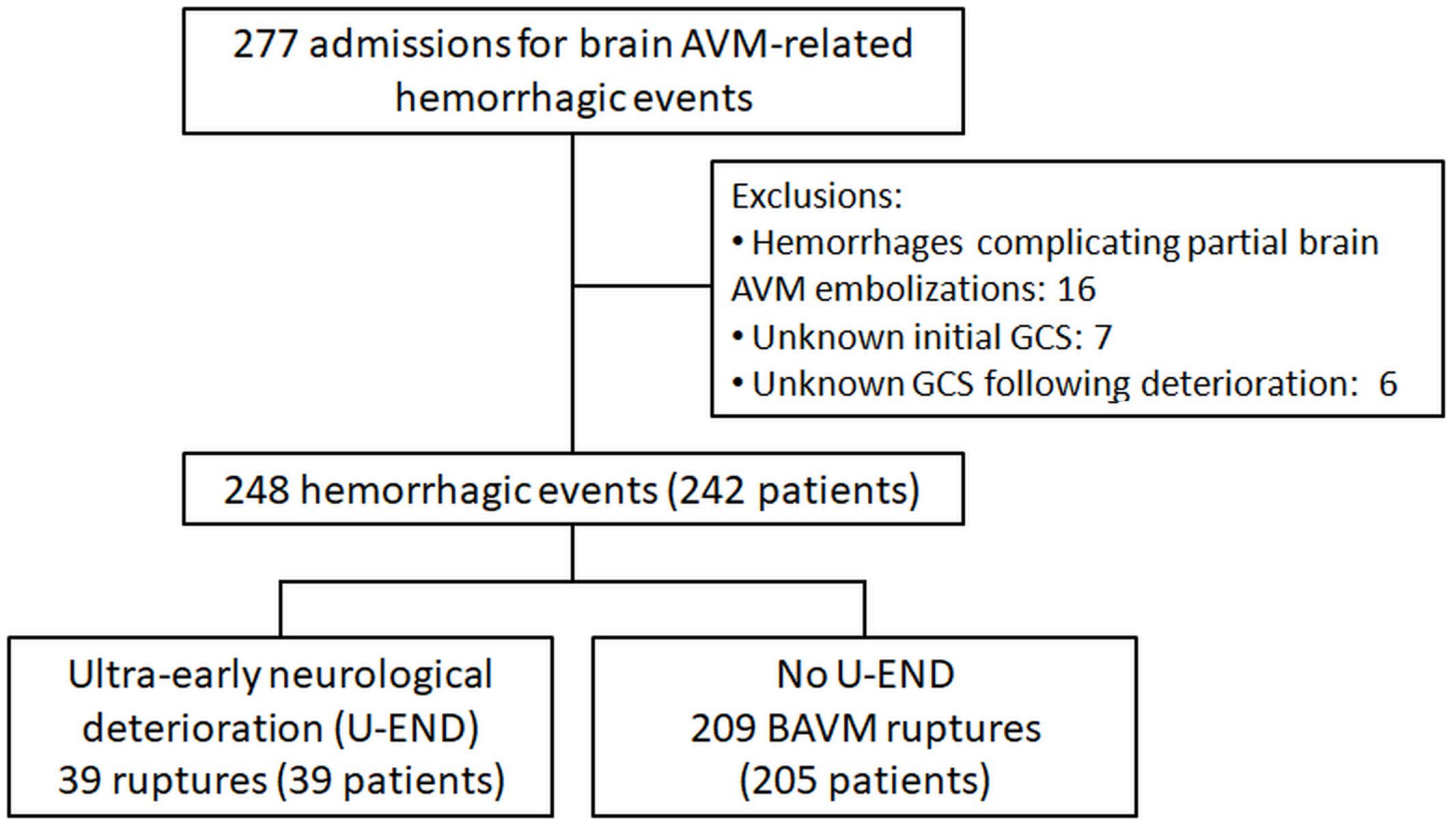
BAVM, Brain arteriovenous malformation; GCS, Glasgow Coma Scale; U-END, Ultra-early neurological deterioration.

In total, 242 patients presenting with 248 BAVM ruptures (six patients included for two independent hemorrhagic occurrences) met the inclusion criteria (Figure 1). Table 1 summarizes the patient’s characteristics. A total of 39 (16%) patients with BAVM ruptures presented with U-END. Out of the six patients included twice for two independent hemorrhagic occurrences, two were included in both U-END and no U-END groups (once with U-END and another without U-END), explaining the discrepancy between the total of patients included (242) and the sum of patients in each of the two subgroups (39 and 205 patients in the U-END and No U-END subgroups, respectively).

**Table 1:** A comparison of brain arteriovenous malformation (BAVM) ruptures with and without ultra-early neurological deterioration (U-END).

	Total study population (248 BAVM ruptures)	U-END (39 BAVM ruptures)	No U-END (209 BAVM ruptures)	*p* value
*Demographics and past medical history*				
Age at admission, years	43.1 ± 15.7	47.7 ± 14.9	42.3 ± 15.7	0.05
Male sex	140 (56.5)	21 (53.8)	119 (56.9)	0.72
Past BAVM rupture	27/246 (11)	3/38 (7.9)	24/208 (11.5)	0.51
Baseline modified Rankin Scale 0	213/245 (86.9)	31/38 (81.6)	182/207 (87.9)	0.29
*Glasgow Coma Scale (GCS) score*				
Median initial GCS	14 (10–15)	14 (12–15)	15 (10–15)	0.89
Median lowest GCS	13 (7–15)	7 (6–11)	14 (10–15)	<10^−3^
*CT scan*				
Intracerebral hemorrhage	199/246 (80.9)	30/38 (78.9)	169/208 (81.2)	0.74
Infratentorial location	45/199 (22.6)	8/30 (26.7)	37/169 (21.9)	0.63
Volume (mL)	36.7 ± 36.6	42 ± 33.2	35.8 ± 37.2	0.39
Intraventricular hemorrhage	153/246 (62.4)	34/38 (89.5)	119/207 (57.5)	<10^−3^
Hydrocephalus	104/245 (42.4)	27/37 (73)	77/208 (37)	<10^−3^
Subarachnoid hemorrhage	60/246 (24.4)	15/38 (39.5)	45/208 (21.6)	0.02
Subdural hemorrhage	20/246 (8.1)	3/38 (7.9)	17/208 (8.2)	0.95
*Biological markers*				
Mean admission (48 h) S100 protein serum level	0.44 ± 0.61	0.35 ± 0.37	0.46 ± 0.65	0.35
Elevated troponin	25/173 (14.5)	5/30 (16.7)	20/143 (14)	0.71
*Inpatient management*				
Surgery	90 (36.3)	16 (41)	74 (35.4)	0.5
Ventricular drain	120/247 (48.6)	30/39 (76.9)	90/208 (43.3)	<10^−3^
Embolization	71 (28.6)	13 (33.3)	58 (27.8)	0.48
Acute phase BAVM obliteration	63 (25.4)	8 (20.5)	22 (26.3)	0.45
*Outcome*				
Duration of sedation (days)	3.4 ± 7.9	9.1 ± 7.7	2.7 ± 7.6	0.001
Duration of stay in intensive care (days)	24.9 ± 24.4	32.6 ± 17	23.2 ± 25.5	0.049
Duration of hospital stay (days)	35.5 ± 34.7	50.2 ± 25.1	32.9 ± 35.6	0.02
Inpatient mortality	27 (10.9)	5 (12.8)	22 (10.5)	0.67
Modified Rankin Scale ≥3 beyond 3 months	87/233 (37.3)	15/38 (39.5)	72/195 (36.9)	0.77

Values are mean ± standard deviation or medians with interquartile range for quantitative variables and percentages for qualitative variables. The denominator is given for binary variables in case of missing data. BAVM, Brain arteriovenous malformation; GCS, Glasgow Coma Scale; and U-END, Ultra-early neurological deterioration.

**Figure 2:**
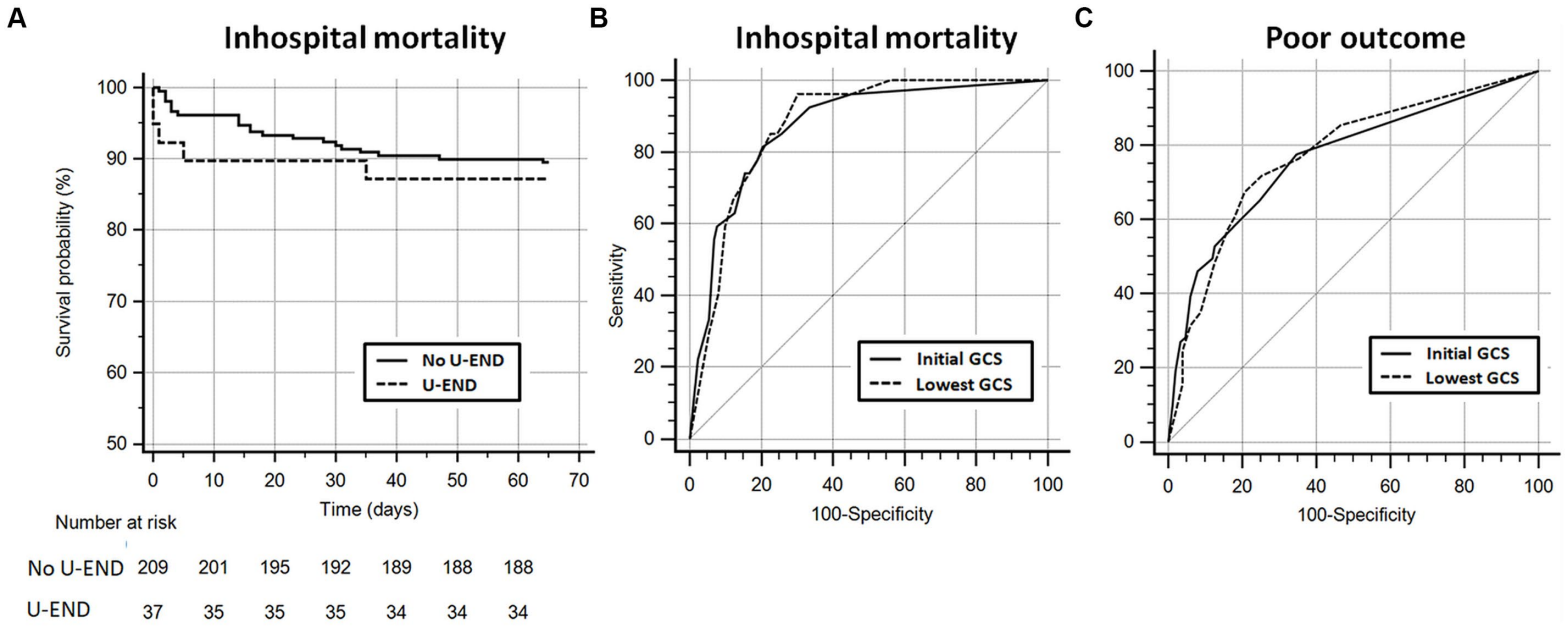
**(A)** Kaplan–Meier survival curve comparing patients with and without ultra-early neurological deterioration (U-END). **(B,C)** Receiver operating characteristic (ROC) curves comparing initial Glasgow Coma Scale (GCS) and lowest early GCS as predictors of in-hospital mortality **(B)** and poor neurological outcome **(C)**. GCS, Glasgow Coma Scale; ROC, Receiver operating characteristic; and U-END, Ultra-early neurological deterioration.

The rate of in-hospital mortality did not differ between BAVM ruptures with (12.8%) and without (10.5%) U-END (*p* = 0.67). In survival analysis (Figures 1, 2A), U-END was not associated with increased in-hospital mortality (hazard ratio = 0.77; 95%CI [0.27–2.2]; *p* = 0.62). In the total study population, the lowest GCS (AUC = 0.88; 95%CI [0.83–0.92]; *p* = 0.83) did not better predict in-hospital mortality when compared to the initial GCS score (AUC = 0.87; 95%CI [0.82–0.91]) (Figure 2B). The outcome at 3 months or beyond was known for 233 cases. The rate of poor outcome did not differ between BAVM ruptures with (39.5%) and without (36.9%) U-END (*p* = 0.77). The lowest GCS (AUC = 0.77; 95%CI [0.72–0.83]; *p* = 0.72) did not better predict poor outcomes when compared to the initial GCS score (AUC = 0.77; 95%CI [0.71–0.82]) (Figure 2C). In contrast, BAVM ruptures presenting with U-END had longer durations of sedation (*p* = 0.001), intensive care stay (*p* = 0.049), and total duration of hospital stay (*p* = 0.02) (Table 1). Exploratory analysis found that U-END patients presented more frequently with IVH (*p* < 10^–3^), hydrocephalus (*p* < 10^–3^), and SAH (*p* = 0.02), leading to the more frequent need for external ventricular drainage (*p* < 10^–3^). In multivariate logistic regression, only an initial GCS of ≤8 (odds ratio (OR) 7.7 [95%CI, 2.2–27.6]; *p* = 0.002) and an S100Bmax48 (OR 9.5 [95%CI, 2.4–37.9]; *p* = 0.002) were predictive of in-hospital mortality. Only GCS of ≤8 (OR 5.6 [95%CI, 2.4–13.1]; *p* < 10^–3^) and an S100Bmax48 (OR 5.4 [95%CI, 2.5–12]; *p* < 10^–3^) were predictive of poor outcome. U-END was not an independent predictor of in-hospital mortality or poor outcomes.

Based on the results of the univariate analysis in Table 1, age at admission, IVH, hydrocephalus, and SAH were included in a multivariate analysis to identify independent determinants of U-END. Only Hydrocephalus (OR 2.6 [95%CI, 1.1–6.4]; *p* = 0.03) and IVH (OR 3.5 [95%CI, 1.1–11.7]; *p* = 0.04) were identified as independent determinants of U-END. When compared to matched controls based on initial GCS, U-END patients presented more frequently with IVH (*p* = 0.009), hydrocephalus (*p* = 0.003), and SAH (*p* = 0.02) (Table 2). The results showed that duration of sedation (*p* = 0.002), stay in intensive care (*p* < 10^–3^), and total hospital stay (*p* < 10^–3^) were longer in U-END patients, without any significant difference in mortality or poor outcome. In contrast, when compared to controls based on the lowest GCS, U-END patients presented with lower early S100B protein serum levels (*p* = 0.02) and less frequent poor outcomes (*p* = 0.03).

**Table 2:** A comparison of brain arteriovenous malformation (BAVM) ruptures presenting with ultra-early neurological deterioration (U-END) with matched controls based on initial or final (lowest) Glasgow Coma Scale (GCS) scores.

	U-END 39 BAVM ruptures)	Initial GCS comparison group (39 BAVM ruptures)	*p* value	Lowest GCS comparison group (39 BAVM ruptures)	*p* value
*Demographics and past medical history*					
Age at admission, years	47.7 ± 14.9	42.8 ± 17.5	0.2	41.1 ± 16.1	0.31
*Glasgow Coma Scale (GCS) score*					
Median initial GCS (IQR)	14 (12–15)	14 (12–15)	1	7 (6–11)	<10^−3^
Mean initial GCS	13 ± 2.6	13 ± 2.6	1	8.2 ± 3.4	<10^−3^
Median lowest GCS (IQR)	7 (6–11)	14 (12–15)	<10^−3^	7 (5–11)	1
Mean lowest GCS	8 ± 3.3	13 ± 2.6	<10^−3^	8 ± 3.4	0.97
*CT scan*					
Intracerebral hemorrhage	30/38 (78.9)	30 (76.9)	0.83	32 (82.1)	0.73
Infratentorial location	8/30 (26.7)	4/30 (13.3)	0.19	11/32 (28.2)	0.47
Volume (mL)	42 ± 33.2	37.7 ± 48.3	0.69	55.2 ± 34.3	0.13
Intraventricular hemorrhage	34/38 (89.5)	24 (64.1)	0.009	29 (74.4)	0.088
Mean Graeb score	6 ± 3	6.2 ± 3.1	0.82	6.3 ± 3	0.68
Hydrocephalus	27/37 (73)	15 (38.5)	0.003	24 (61.5)	0.29
Subarachnoid hemorrhage	15/38 (39.5)	6 (15.4)	0.02	11 (28.2)	0.3
BNI score ≥ 3	6/15 (40)	2 (33)	0.78	2 (18.2)	0.24
Subdural hemorrhage	3/38 (7.9)	3 (7.7)	0.97	1 (2.6)	0.3
*Biological markers*					
Mean admission (48 h) S100 protein serum level	0.35 ± 0.37	0.35 ± 0.41	0.98	0.83 ± 1	0.01
Elevated troponin	5/30 (16.7)	2/26 (7.7)	0.32	7/33 (21.2)	0.65
*Outcome*					
Duration of sedation (days)	9.1 ± 7.7	2.6 ± 5.5	0.002	9.4 ± 14.6	0.94
Duration of stay in intensive care (days)	32.6 ± 17	15.9 ± 12.5	<10^−3^	28.3 ± 16	0.33
Duration of hospital stay (days)	50.2 ± 25.1	25.6 ± 21.2	<10^−3^	43.8 ± 29.6	0.43
Inpatient mortality	5 (12.8)	4 (10.3)	0.72	11 (28.2)	0.09
mRS ≥ 3 beyond 3 months	15/38 (39.5)	12/37 (32.4)	0.53	24/37 (64.9)	0.03

Values are mean ± standard deviation (SD) or medians with interquartile range (IQR) for quantitative variables and percentage for qualitative variables. The denominator is given for binary variables in case of missing data. BAVM, Brain arteriovenous malformation; GCS, Glasgow Coma Scale; mRS, Modified Rankin scale; and U-END, Ultra-early neurological deterioration.

## 4 Discussion

Ultra-early neurological deterioration in the study population of patients admitted following BAVM rupture did not result in increased mortality or poor outcome and was most often related to IVH and hydrocephalus. With the exception of hydrocephalus, hemorrhage severity and prognosis were more determined by the initial rather than the lowest GCS in U-END patients with BAVM rupture.

Decisions regarding the most appropriate level of care in neurocritically ill patients are partially based on outcome prognostication, which in turn is related to prognostic markers (4). Caution should, however, be exercised in clinical practice when handling early prognostic markers of poor outcomes, as overconfidence can lead to a self-fulfilling prophecy. This consideration is particularly important in the context of neurocritically ill patients who often die following withdrawal of life-sustaining treatment (15). The GCS is a strong prognostic marker following both ICH in general and hemorrhage secondary to BAVM rupture in particular (4, 8).

Neurological deterioration following a stroke can have a number of causes, depending on stroke type, including and not exhaustively, hematoma expansion, seizure, or obstructive hydrocephalus. Exploratory analysis of the randomized Field Administration of Stroke Therapy-Magnesium (FAST-MAG) trial has found U-END to occur in 30.8% of ICH patients, nearly twice the figure found in the context of BAVM rupture (1). More importantly, Shkirkova et al. found a more than four-fold increase in poor neurological outcomes and a three-fold increase in mortality in ICH patients presenting with U-END (1). U-END following BAVM rupture did not result in increased mortality or poor outcome in this study, possibly because the mechanism of U-END in this context is hydrocephalus-related intracranial hypertension, which is reversible through external ventricular drainage. BAVM ruptures with U-END tend to have hemorrhage severity and prognostic profiles closer to controls matched based on their initial rather than the lowest GCS, except for durations of hospital stay, which may be prolonged because of intraventricular hemorrhage and external ventricular drainage, although this remains speculative. S100B protein is a biochemical marker of secondary neurological complications in neurocritical care patients (13). Early elevation of S100B protein serum level has been demonstrated to be strongly associated with in-hospital mortality after BAVM rupture (14). When compared to controls based on the lowest GCS, U-END patients in this study presented with lower early S100B protein serum levels, indicative of a less severe hemorrhagic profile, confirmed by better neurological outcomes.

The study is retrospective and monocentric, and all the usual caveats apply. Indeed, several patients were excluded because of unknown initial GCS scores. More importantly, patients known to have deteriorated early before or upon admission but for which the extent of deterioration was not recorded were excluded. The data presented in this study has to be replicated in a prospective population where such loss of information can easily be mitigated. Another potential source of U-END may be rebleeding, which would not have been diagnosed, given that deterioration mostly occurs by definition before imaging. However, it is the author’s opinion that rebleeding is a minor contributor to U-END in the setting of BAVM rupture. Indeed, previous studies have shown early rebleeding to be a rare event following BAVM rupture, and ultra-early rebleeding is consequently expected to be exceptional (16). Finally, the study may be underpowered to demonstrate the impact of U-END on the outcome following BAVM rupture. This raises the need for external multicentric replication of the results. In particular, in-hospital mortality is a rare event, and the study may be underpowered to detect a modest or moderate impact of U-END on this specific outcome.

## 5 Conclusion

Ultra-early neurological deterioration in ruptured BAVMs was most often related to IVH and hydrocephalus and did not result in increased mortality or poor outcomes in the study population. Based on the results of this study, in ruptured BAVM patients with U-END, prognostication should be based on the initial GCS rather than the lowest GCS, especially when there is no obvious cause of deterioration other than hydrocephalus.

## Data availability statement

The raw data supporting the conclusions of this article will be made available by the authors, without undue reservation.

## Ethics statement

The studies involving humans were approved by the CERIM (CERF Interface Recherche Bioéthique Institutional Review Board) ethical review board approved this study (approval number CRM-201-119). The studies were conducted in accordance with the local legislation and institutional requirements. The ethics committee/institutional review board waived the requirement of written informed consent for participation from the participants or the participants’ legal guardians/next of kin because the need for patients’ informed consent was waived given the retrospective design.

## Author contributions

ES: Conceptualization, Data curation, Formal analysis, Investigation, Methodology, Supervision, Validation, Writing – original draft, Writing – review & editing. P-MC: Data curation, Writing – review & editing. IH: Data curation, Writing – review & editing. JC: Data curation, Writing – review & editing. AJ: Data curation, Writing – review & editing. LG: Data curation, Writing – review & editing. JA: Data curation, Writing – review & editing. ME: Data curation, Writing – review & editing. CA: Data curation, Writing – review & editing. BM: Conceptualization, Writing – review & editing. AN: Conceptualization, Writing – review & editing. KP: Conceptualization, Writing – review & editing. SL: Conceptualization, Writing – review & editing. N-AS: Conceptualization, Writing – review & editing. VD: Conceptualization, Data curation, Supervision, Writing – review & editing. FC: Conceptualization, Data curation, Supervision, Writing – review & editing.
